# Visualisation of proteome-wide ordered protein abundances in *Trypanosoma brucei*

**DOI:** 10.12688/wellcomeopenres.17607.2

**Published:** 2023-02-01

**Authors:** Michele Tinti, Michael A. J. Ferguson

**Affiliations:** 1 Wellcome Centre for Anti-Infectives Research, School of Life Sciences, University of Dundee, Dundee, DD1 5HN, UK

**Keywords:** Trypanosoma brucei, proteomics, bloodstream form, procyclic form, quantification, iBAQ, web application

## Abstract

**Background:** *Trypanosoma brucei *is a protozoan parasite and etiological agent of human and animal African trypanosomiasis. It has a complex life cycle, but the most studied cellular types are the *in vitro* cultivated bloodstream- and procyclic-forms. These correspond to the replicating, mammalian host bloodstream-dwelling, slender trypomastigotes and tsetse vector midgut-dwelling procyclic lifecycle stages, respectively. Several proteomics studies have reported the differential abundance of proteins between these *in vitro *cultivated cell types. However, there are no datasets providing protein abundance, from most to least abundant, within and between both cell types.

**Methods: **We used MaxQuant software 1.6.10.4 to reprocess a recent large-scale proteomics experiment from our laboratory and extracted intensity-based quantifications of the bloodstream and procyclic form proteomes.

**Results: **We created a web interface to visually explore protein abundances within and between the *in vitro* cultivated *T. brucei* bloodstream and procyclic form proteomes.

**Conclusions: **The protein abundance visualization tool, searchable by protein name(s) and attribute(s), is likely to be useful to the trypanosome research community. It will allow users to contextualise their proteins of interest in terms of their abundances in the *T. brucei* bloodstream and procyclic form proteomes.

## Introduction

The protozoan parasite *Trypanosoma brucei* is transmitted to its human and animal hosts by the tsetse fly ( *Glossina* species), which is found only in sub-Saharan Africa ^1^. The parasites replicate as the procyclic form (PCF) in the tsetse midgut. Some of these differentiate into the replicating epimastigote form as they migrate to the tsetse salivary glands. These differentiate into non-dividing metacyclic trypomastigote forms that are adapted for transmission to the mammalian host during a tsetse bloodmeal. Once in the host, the parasites differentiate into replicating slender bloodstream form (BSF) trypomastigotes and some of these further differentiate into non-dividing stumpy forms that are adapted for transmission to the tsetse vector ^1^. If left untreated, the parasites invade the human central nervous system causing character disintegration, coma and death ^1^. The proteomes of *in vitro* cultivated BSF and PCF cells have been analysed quite extensively ^2– 5^; however, the focus of such studies has been the determination of the differential protein abundances between the two lifecycle forms rather than ranked-order relative protein abundance values. The latter can be particularly useful when assessing protein functions. With this in mind, we decided to re-process a deep proteome study recently published in our laboratory ^6^ to extract **i**ntensity- **b**ased **a**bsolute **q**uantification (iBAQ) ^7^ values for the *in vitro* cultivated BSF and PCF lifecycle stages. The iBAQ method estimates protein abundances in a complex proteome by integrating all the peptide intensities measured by mass spectrometry for each detected protein group and dividing them by the number of theoretical observable tryptic peptides (i.e., between six and 30 amino acids) contained within them.

## Methods

We reprocessed with MaxQuant software 1.6.10.4 ^8^ the protein turnover dataset of *T. brucei* described in Tinti *et al*., 2019 ^6^ deposited at the PRIDE database ^9^ with accession number: PXD007115. We used the same parameters described in 6 except for: (i) Using the protein sequences version 46 for the TREU927 clone and version 52 for the Lister427_2018 clone, downloaded from TriTrypDB ^10^. (ii) The protein sequences of the TREU927 clone were also supplemented with the BES1/TAR40 protein sequences downloaded from NCBI (accession number: FM162566). (iii) The iBAQ option in the MaxQuant software was selected.

The Tinti *et al*., 2019 paper ^6^ reported the *T. brucei* Lister strain 427 *in vitro* cultivated BSF and PCF protein half-lives computed from a label-chase experiment using a **S**table **I**sotope **L**abeling using **A**mino acids in **C**ell culture (SILAC) approach ^11^. BSF and PCF parasites were labelled to steady-state in medium SILAC culture media (M) and chased into light SILAC culture media (L). The experiment consisted of seven and nine time points for the BSF and PCF samples, respectively, with three biological replicates each. The samples of the time course experiments were also mixed 1:1 with BSF or PCF parasite lysates, as appropriate, labelled to steady-state in heavy SILAC culture media (H) to provide an internal standard for normalisation. Total proteomes from each biological replicate time point were digested with trypsin and the resulting peptides were separated into 10 sub-fractions for LC-MS/MS analysis. The total dataset therefore represents a total of 210 and 270 individual LC-MS/MS runs for the BSF and PCF samples, respectively, providing particularly deep and robust proteomes. After data re-processing with MaxQuant ^8^ we considered only the iBAQ values of the H labelled samples retrieved from the proteinGroups.txt file. After equalizing the median values of all the replicates, any missing values were replaced by sampling from a random distribution centred around the minimum value. Finally, the median iBAQ values of the heavy-labelled peptides for each protein were taken for the BSF and PCF replicates. We also computed the numerical data ranks from the median iBAQ values starting from 1 (the least abundant) to n (the most abundant) where n is the number of the protein group identified. The gene id of the leading protein of the protein groups assembled by MaxQuant ^8^ was used to report the gene id description. The script to process the data were developed in Jupyter Notebook version 0.5.1 using the Python SciPy version 1.4.1 ecosystem ^12^.

### Implementation

**Figure 1.**
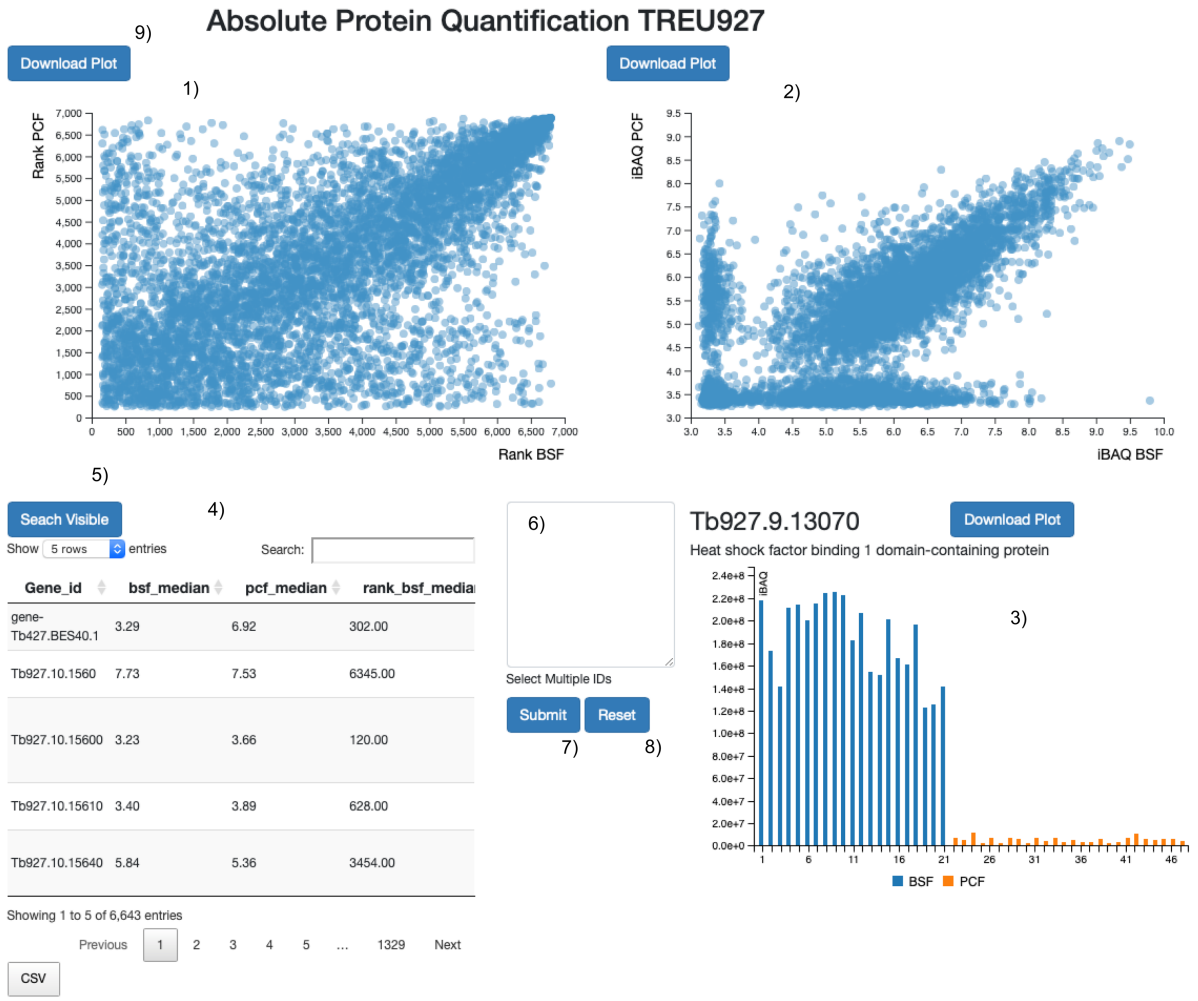
Web application layout. Screen shot of the web application user interface. 1) The protein abundance rank positions, from least to most abundant (1 – >7,000) according to iBAQ values (blue circles). These are plotted for the bloodstream form (x-axis) and the procyclic form (y-axis) proteomes. 2) The median protein iBAQ values themselves (blue circles) are plotted for the bloodstream form (x-axis) and the procyclic form (y-axis) proteomes. 3) Bar plot of all of the individual iBAQ values from the bloodstream form (blue) and procyclic form (amber) proteomes used to determine the median iBAQ values. 4) The main search table that reports the gene id of the leading protein id of the protein group (Gene id); the median iBAQ value in the bloodstream form (bsf_median) and procyclic form (pcf_median) proteomes; the median iBAQ values, rank transformed, in the bloodstream form (rank_bsf_median) and procyclic form (rank_bsf_median) proteomes; the gene id description (Desc); The protein ids of the protein group (Protein IDs). 5) One click of the button and all the proteins visible in the search table [4] are highlighted in the scatter plots [1 and 2]. 6) Text input area to search for gene ids in the two scatter plots [1 and 2]. 7) On click of the button the gene ids present in 8 are highlighted in the two scatter plots [1 and 2]. 8) On click of the button the two scatter plots [1 and 2] are reset. 9) The download button allows the user to save locally a scalable vector graphic (SVG) image of the plot.

We reprocessed a large proteomics dataset of *T. brucei* to extract iBAQ values and ranked the proteins by their abundance values. We focused on two of the most used reference protein databases of *T. brucei*; the TREU927 clone that is better annotated with respect to other clones and the Lister 427 clone that is the most used in laboratory experiments and which was the source of the original proteomics data ^6^. We then assembled a web application to compare the iBAQ and protein rank values extracted from the proteomics data for the PCF and BSF lifecycle stages ( Figure 1). A tour of the web application starts on page loading and guides the user to the main functionalities. Briefly, we provided two interactive scatter plots, one for the rank values and one for the iBAQ values ( Figure 1). Also, an interactive data table allows the data to be searched using protein names or descriptions ( Figure 1). Finally, we coded a bar plot of the normalized iBAQ values without data imputation to visually assess: (i) Data reproducibility and (ii) The number of data points used to compute the iBAQ medians ( Figure 1). We implemented two identical web applications to visualise the protein abundance rank in BSF and PCF lifecycle stages, except that one is based on the TREU927 proteome and the other on the Lister427_2018 proteome. The source code of the web application was developed in JavaScript D3 version 5 and JavaScript C3 version 0.7.20 for the scatter plots and bar plot visualisation, and DataTables v1.10.21 for the rendering of the data in tabular format. Custom JavaScript code was used to make the scatterplots, bar plot and the data table responsive with respect to each other and to the user interactions.

### Operation

The websites are hosted at Cloudflare Pages: https://tbrucei-ibaq-927.pages.dev/ for the TREU927 proteome and https://tbrucei-ibaq-427.pages.dev/ for the Lister 427_2018 proteome. It should be relatively straightforward to clone the git repository (git clone mtinti/tbrucei_ibaq) and run the application locally by opening the application folder with Microsoft's Visual Studio Code software (version 1.63.1) with the “ golive” plugin (version 5.7.2). We recommend 1.6 GHz or faster processor and 1 GB of RAM.

### Use cases

The user is presented with a responsive web application with four main components: two scatter plots ( Figure 1.1 and 1.2), one bar plot ( Figure 1.3) and a search table ( Figure 1.4). The user can search for a protein id or attribute (for example, “mitochondrial”) in the table search field, and the table will display the matched results. Hovering on the table rows ( Figure 1.4) will highlight the position of the proteins in the two linked scatter plots ( Figure 1.1 and 1.2) and pull the iBAQ values into the bar plot ( Figure 1.3). Any protein visible in the search table ( Figure 1.4) will be visualized at once in the two linked scatter plots ( Figure 1.1 and 1.2) by clicking the “search visible” button ( Figure 1.5). Multiple protein ids can be pasted into the text search field ( Figure 1.6) and similarly visualized at once in the two linked scatter plots ( Figure 1.2 and 1.3) by clicking the “submit” button ( Figure 1.7). The scatter plot visualisations can be reset by clicking on the “reset” button ( Figure 1.8).

The user can hover over any of the circles in the scatter plots ( Figure 1.2 and 1.3) to visualize further information on the proteins they represent, such as the protein id, protein description and the x and y iBAQ or protein abundance rank order values in BSF and PCF cells, respectively. Any protein hovered over will be highlighted in both scatter plots ( Figure 1.2 and 1.3) and in the search table ( Figure 1.4), and the user will see the corresponding iBAQ values in the bar plot ( Figure 1.3). By clicking on any protein circle, the user can annotate it with custom text, with the gene id presented as the default option. The annotation can be moved around to better fit in the scatter plot area.

The user can drag a rectangle on either of the two scatter plots ( Figure 1.2 and 1.3) to activate a zoom functionality. Only the circles contained in the dragged area will be visualised in the scatter plot and in the search table. The user can download the scatter plots and bar plot as Scalable Vector Graphics (SVG) by clicking on the “download plot” button ( Figure 1.9).

## Conclusions

We present an effort to rank the *T. brucei* proteome by absolute abundance using the iBAQ values and we provided a visualisation tool to explore the data ( Figure 1). The iBAQ values have been shown to perform reasonably well to determine absolute abundance but it is dependent on the quality of the peptide ionization and the number of peptides identified during the mass spectrometry analysis ^7, 13^. The iBAQ and other quantification methods use a data acquisition protocol in mass-spectrometry named data-dependent acquisition (DDA) ^14^. Recent advances in another type of data acquisition in mass spectrometry, named data-independent acquisition (DIA), promise to increase the number of quantified peptides and consequently improve the protein quantification ^15^. It is likely that in the future we will replace our estimates of protein abundance with a dataset originating from DIA experiments, but for the time being we believe that our strategy provides a good and useful approximation.

## Data Availability

No data are associated with this article. PRIDE Project: Proteome turnover in bloodstream and procyclic form *Trypanosoma brucei* measured by quantitative proteomics. Accession number PXD007115; https://identifiers.org/pride.project:PXD007115 TriTrypDB: Proteome of *Trypanosoma brucei* procyclic form mitochondrial enriched fraction. Accession number TREU927; https://identifiers.org/tritrypdb:TREU927 TriTrypDB: ChIP-Seq of H4K10ac, bromodomain protein (BDF3), and four histone variants mark the boundaries of polycistronic transcription units in *Trypanosoma brucei*. Accession number TBLister 427-2018; https://identifiers.org/tritrypdb:TbLister 427-2018 NCBI Protein: *Trypanosoma brucei* Lister 427 surface glycoprotein expression site BES1/TAR40 from bloodstream. Accession number FM162566; https://identifiers.org/ncbiprotein:FM162566

## Software availability

Source code available from: https://github.com/mtinti/tbrucei_ibaq including analysis pipeline, links to the raw data and code used to extract the data for the web applications. The code to reproduce the data extraction form the protein group files can be inspected using the mybinder badge in GitHub.

Archived source code at time of publication: Zenodo: mtinti/tbrucei_ibaq: v0.2 add zenodo. https://doi.org/10.5281/zenodo.5526824 ^16^

This project contains the following data:

427

- proteinGroups_ibaq.txt.gz (output file of MaxQuant for the lister 427_2018 clone)

927

- proteinGroups_ibaq.txt.gz (output file of MaxQuant for the TREU927 clone)

Anti-infectives-RGB_200pc.png

README.md (GitHub file describing the repository)

analysis_427.ipynb (The code to extract the data for the lister 427_2018 clone)

analysis_927.ipynb (The code to extract the data for the TREU927 clone)

postBuild (configuration file for mybinder)

requirements.txt (python packages used to extract the data from the protein group files)

utilities.py (python functions used to extract the data from the protein group files)

web_data

- E427 (the web application code for the lister 427_2018 clone)
- E927 (the web application code for the TREU927 clone)

Licence: MIT
