# Supplementary material for: Visualisation of proteome-wide ordered protein abundances in Trypanosoma brucei
Source: Wellcome Open Res. 2023 Feb 1;7:34. Originally published 2022 Jan 31. [Version 2] doi: 10.12688/wellcomeopenres.17607.2 (PMC8889043; doi:10.12688/wellcomeopenres.17607.2)
Supplement: Peer review history

# Peer review: Reviewer response for version 1

**DOI:** 10.21956/wellcomeopenres.19475.r48676

**Published:** 2022-03-15

**Authors:** Kent L. Hill

**Affiliations:** 1 Department of Microbiology, Immunology, and Molecular Genetics, University of California Los Angeles, Los Angeles, CA, USA

The authors provide a 'reprocessing' of published deep proteome datasets from PCF and BSF *Trypanosoma brucei*, allowing determination of relative protein abundance for each protein identified in a given life cycle stage. This is valuable information not readily accommodated by existing proteomic datasets for this organism. The authors further provide a user-friendly navigation tool that allows users to readily identify proteins based on relative abundance in PCF or BSF stages, as well as search for individual proteins (using geneID or description) to quickly obtain relative protein abundance for the protein of interest. The datasets and navigation tools are available for both the TREU927 and the Lister 427 clones of *T. brucei *(a feature that I expect will be much appreciated by many users).

 These are useful data and very useful and informative data navigation tools. I expect this will be a valuable and oft-used resource for the research community.

 One request for the paper is to adjust the position of the scatter plots in the manuscript figure (Fig 1.1 and 1.2, currently with 'Rank' plot on the left and 'iBAQ' on the right) to match their position in the online tool (currently 'Rank' plot on the right and 'iBAQ' on the left). Not critical, but the different layout is cause for brief confusion if alternating between manuscript and online tool.

Are the conclusions about the tool and its performance adequately supported by the findings presented in the article?

Yes

Is the rationale for developing the new software tool clearly explained?

Yes

Is the description of the software tool technically sound?

Yes

Are sufficient details of the code, methods and analysis (if applicable) provided to allow replication of the software development and its use by others?

Yes

Is sufficient information provided to allow interpretation of the expected output datasets and any results generated using the tool?

Yes

Reviewer Expertise:

Molecular Parasitology, Cell Biology

I confirm that I have read this submission and believe that I have an appropriate level of expertise to confirm that it is of an acceptable scientific standard.

# Response

**Published:** 2022-03-24

**Authors:** Michele Tinti

**Affiliations:** Dundee University, UK

We thank the reviewer for the feedback. I recently swiched the plots for coding reasons and I also added some new utilities. I will describe these changes in the next paper versions.

# Peer review: Reviewer response for version 1

**DOI:** 10.21956/wellcomeopenres.19475.r48579

**Published:** 2022-03-01

**Authors:** Stefan Magez

**Affiliations:** 1 Laboratory for Biomedical Research, Ghent University Global Campus, Incheon, South Korea; 2 Laboratory for Cellular and Molecular Immunology, Vrije Universiteit Brussel, Brussels, Belgium

This is going to be a very useful tool for many people with many different interests, including people in the field of immunoparasitology trying to understand how the host context changes protein expression in the parasite and for example how the host immune system is involved in the process of regulation the quorum sensing machinery of trypanosomes. Many more ideas will come to mind as people start to 'play' with this tool. 

 One suggestion:

- As some technical glitches will come to the surface while people are using the tool, it will be important to provide the user with a contact to whom to direct very practical questions (the corresponding author/PI might not be the person who will be able to free time to deal with this...)

In all, very impressed with the initiative! And a very welcome gift to the trypanosome research community.

Are the conclusions about the tool and its performance adequately supported by the findings presented in the article?

Yes

Is the rationale for developing the new software tool clearly explained?

Yes

Is the description of the software tool technically sound?

Yes

Are sufficient details of the code, methods and analysis (if applicable) provided to allow replication of the software development and its use by others?

Yes

Is sufficient information provided to allow interpretation of the expected output datasets and any results generated using the tool?

Yes

Reviewer Expertise:

Immunoparasitology, diagnostic development and vaccine research - focused on trypanosomosis.

I confirm that I have read this submission and believe that I have an appropriate level of expertise to confirm that it is of an acceptable scientific standard.

# Response

**Published:** 2022-03-04

**Authors:** Michele Tinti

**Affiliations:** Dundee University, UK

We indeed hope that this tool will be useful for the T. brucei community. We will add a mail contact to report issues with the application.

# Peer review: Reviewer response for version 1

**DOI:** 10.21956/wellcomeopenres.19475.r48550

**Published:** 2022-02-08

**Authors:** Vyacheslav Yurchenko

**Affiliations:** 1 Life Science Research Centre, Faculty of Science, University of Ostrava, Ostrava, Czech Republic

The presented work of Tinti & Ferguson reports visualization of proteome-wide ordered protein abundances in *T. brucei*. I should mention that I noticed and read this paper even before I was asked to review it. My overall impression of the newly developed tool is that it is destined to become a standard part of any serious gene-function analysis in *Trypanosoma brucei*. On a side note, I particularly liked that "little tour" offered by the program. I do have some questions and suggestions on how it might be improved. 

1. Would it be possible to add and visualize proteomic data from other datasets (not only from Tinti *et al. *2019)? I envision that one resource combining all the available data may be an ultimate goal. 
2. I am slightly confused by the note about BES1/TAR40 locus (~ 60 kb, GenBank  FM162566). Why did you supplement TREU927 dataset by LISTER427 sequence? 
3. This did not work when I tried it: "The user can drag a rectangle on either of the two scatter plots (Figure 1.2 and 1.3) to activate a zoom functionality. Only the circles contained in the dragged area will be visualised in the scatter plot and in the search table."
4. The provided netlife links (on page 3) redirected me to other web-addresses. Please replace them with direct links.    

Are the conclusions about the tool and its performance adequately supported by the findings presented in the article?

Yes

Is the rationale for developing the new software tool clearly explained?

Yes

Is the description of the software tool technically sound?

Yes

Are sufficient details of the code, methods and analysis (if applicable) provided to allow replication of the software development and its use by others?

Yes

Is sufficient information provided to allow interpretation of the expected output datasets and any results generated using the tool?

Yes

Reviewer Expertise:

Molecular parasitology, omics

I confirm that I have read this submission and believe that I have an appropriate level of expertise to confirm that it is of an acceptable scientific standard.

# Response

**Published:** 2022-02-09

**Authors:** Michele Tinti

**Affiliations:** Dundee University, UK

1)Indeed, it is possible. We find it convenient to use this tool to visualise differential expression (RNA-seq) or abundance (proteomics) analysis using volcano and MA plots. However, creating a resource to display all the available data is beyond our reach. We will contact the TriTrypDB database and ask if they will consider implementing this visualisation for the datasets deposited there. 

 2)The 427 strain is commonly used for experiments in the lab, and the BES1 locus is available for the T. brucei 427 strain. However,  the BES1 locus is absent in the sequenced genome of the T. brucei 927 strain that is commonly used for data analysis, as it provides a better gene annotation. 

 3)I tried the functionality in Firefox, Chrome and Safari using a mac laptop. I suspect it might be a windows system issue. I will do more tests to find the problem.

 4) We will ask the publisher to change the links of the apps if possible. Unfortunately, I had to change the web hosting company after submitting the paper for an issue I didn't expect.
